# Precision Phenotyping Reveals Novel Loci for Quantitative Resistance to Septoria Tritici Blotch

**DOI:** 10.34133/2019/3285904

**Published:** 2019-09-29

**Authors:** Steven Yates, Alexey Mikaberidze, Simon G. Krattinger, Michael Abrouk, Andreas Hund, Kang Yu, Bruno Studer, Simone Fouche, Lukas Meile, Danilo Pereira, Petteri Karisto, Bruce A. McDonald

**Affiliations:** ^1^Molecular Plant Breeding, Institute of Agricultural Sciences, ETH Zurich, Zurich, Switzerland; ^2^Plant Pathology, Institute of Integrative Biology, ETH Zurich, Zurich, Switzerland; ^3^Biological and Environmental Science and Engineering Division, King Abdullah University of Science and Technology (KAUST), Thuwal, Saudi Arabia; ^4^Crop Science, Institute of Agricultural Sciences, ETH Zurich, Zurich, Switzerland

## Abstract

Accurate, high-throughput phenotyping for quantitative traits is a limiting factor for progress in plant breeding. We developed an automated image analysis to measure quantitative resistance to septoria tritici blotch (STB), a globally important wheat disease, enabling identification of small chromosome intervals containing plausible candidate genes for STB resistance. 335 winter wheat cultivars were included in a replicated field experiment that experienced natural epidemic development by a highly diverse but fungicide-resistant pathogen population. More than 5.4 million automatically generated phenotypes were associated with 13,648 SNP markers to perform the GWAS. We identified 26 chromosome intervals explaining 1.9-10.6% of the variance associated with four independent resistance traits. Sixteen of the intervals overlapped with known STB resistance intervals, suggesting that our phenotyping approach can identify simultaneously (i.e., in a single experiment) many previously defined STB resistance intervals. Seventeen of the intervals were less than 5 Mbp in size and encoded only 173 genes, including many genes associated with disease resistance. Five intervals contained four or fewer genes, providing high priority targets for functional validation. Ten chromosome intervals were not previously associated with STB resistance, perhaps representing resistance to pathogen strains that had not been tested in earlier experiments. The SNP markers associated with these chromosome intervals can be used to recombine different forms of quantitative STB resistance that are likely to be more durable than pyramids of major resistance genes. Our experiment illustrates how high-throughput automated phenotyping can accelerate breeding for quantitative disease resistance.

## 1. Introduction

Genome-wide association studies (GWAS) provide a powerful approach to identify genetic markers associated with important quantitative traits in crops (e.g., [1, 2]). The single nucleotide polymorphism (SNP) markers significantly associated with a trait in the GWAS can be directly used in breeding programs for marker-assisted selection or genomic selection and also as tools to enable map-based cloning of the corresponding genes underlying quantitative traits.

An abundant supply of SNP genetic markers is now available for most crops as a result of rapid advances in sequencing technologies. Because phenotyping technologies have not developed as quickly as genotyping technologies, the ability to generate accurate and reproducible phenotypes for quantitative traits is now the primary limitation to progress in breeding for favorable traits [3, 4], including resistance to pests and pathogens [5]. Many research groups are working to develop automated/semiautomated and high-throughput phenotyping of important traits under field conditions, with some reports of success [5–7], but we remain far from the goal of using automated phenotyping to speed progress in plant breeding for useful traits.

Septoria tritici blotch (STB), caused by the fungus *Zymoseptoria tritici,* is currently the most damaging leaf disease on wheat in Europe [8] and is one of the most significant diseases on wheat around the world [9]. *Z. tritici* has a mixed reproductive system, producing airborne ascospores through sexual reproduction that can be disseminated over distances of several kilometers and asexual conidia that are splash-dispersed over spatial scales of only 1-2 meters during the course of a growing season [10]. *Z. tritici* populations are highly variable within fields as a result of its mixed reproductive system, large effective population sizes, and high levels of gene flow among populations [10, 11]. These properties provide a high evolutionary potential that leads to rapid development of virulence against resistant cultivars [10, 12] as well as resistance to fungicides [10, 13, 14]. STB in Europe is controlled mainly by applying fungicides costing over $1 billion per year [15], but many European *Z. tritici* populations have now evolved sufficiently high levels of resistance that fungicides are losing their efficacy [16, 17]. The European Union is planning to ban many fungicides in the near future (EU Regulation 1107/2009). These developments have stimulated new efforts to increase STB resistance through plant breeding.

Many studies have identified strain-specific STB resistance genes that could prove useful in breeding programs (summarized in [18]). These genes are typically identified and mapped by inoculating a single strain of *Z. tritici* onto a segregating population derived from a cross between two parents that differ in their resistance to that strain, with disease ratings often made on seedlings under greenhouse conditions. STB resistance in the field is mainly quantitative, but some examples of major gene resistance were identified (e.g., *Stb6*) that were recently shown to follow the gene-for-gene (GFG) pattern of inheritance [19, 20]. Unfortunately, major STB resistance genes such as *Stb6* typically failed within 3-4 years of deployment as a result of pathogen evolution [12]. A different breeding approach that is expected to slow pathogen evolution and be more durable is to make pyramids of quantitative resistance (QR) genes with additive effects [21, 22]. This approach requires the identification and deployment of QR that is effective across a broad cross section of the *Z. tritici* population as opposed to major gene resistance that works against only a small fraction of the strains found in natural field populations.

Identification of QR is difficult for most pathogens for many reasons including (1) measurement error associated with visual assessments of disease, (2) inherent differences in disease measurements conducted by different people, (3) differences in expression of QR in different environments, (4) the phenotypic effect associated with a QR locus (e.g., 5-10% reduction in disease) which can be too small to detect using disease ratings that operate in larger increments (e.g., 0-9 scales that differentiate disease increments of 10%), and (5) the occurrence of mixed infections by several pathogens under typical field conditions, with overlapping symptoms that often cannot be teased apart (e.g., STB symptoms look very similar to the symptoms associated with tan spot and stagonospora nodorum leaf blotch). These factors combine to create a low heritability for QR that slows progress in accumulating different sources of QR in breeding programs.

The recent development of automated image analysis for STB enabled rapid acquisition of large datasets, including millions of phenotype datapoints that were highly informative under both greenhouse and field conditions, and facilitated the cloning of genes encoding several avirulence effectors, including *AvrStb6* [20] and *Avr3D1* [23, 24], as well as the *Zmr1* gene affecting melanization of *Z. tritici* colonies and pycnidia [25, 26]. We took advantage of the high levels of fungicide resistance in Swiss populations of *Z. tritici* by using fungicide treatments to eliminate competing pathogens in a replicated field experiment [16]. The fungicide treatments enabled a pure-pathogen read-out of quantitative resistance to STB caused by a genetically diverse, natural population of *Z. tritici* in an epidemic that developed under natural field conditions. Here, we use this extensive phenotype dataset in the GWAS to identify 26 chromosome intervals associated with quantitative STB resistance in a broad panel of 335 elite European winter wheat cultivars. Many of these intervals explained 6%-10% of the variance for the associated resistance trait. Several of the intervals contained a relatively small number of annotated genes, including genes known to be associated with disease resistance in wheat or other plants. There was a significant enrichment (*P* < 0.0001) for genes encoding putative receptor kinases and kinases within the 17 chromosome segments spanning less than 5 Mbp. Other candidate genes for STB resistance encoded NB-LRR proteins, F-box LRR proteins, sugar transporters, an ABC transporter, superoxide dismutase, and a TCP transcription factor, illustrating how automated image analysis can lead to identification of plausible candidate genes for quantitative disease resistance.

## 2. Materials and Methods

335 European winter wheat cultivars chosen from the GABI wheat panel [27] were grown in 1.1 × 1.4m plots replicated twice as complete blocks at the Field Phenotyping Platform of the ETH research station in Lindau, Switzerland (47.449°N, 8.682°E, 520 masl) [28]. The plots received full agrochemical inputs typically associated with intensive wheat cultivation in Europe, including mineral fertilizers, a stem shortener, and several pesticide applications. Among the pesticides, fungicides comprising five different active ingredients with three modes of action were applied at three time points over the growing season. Additional details associated with the field experiment are given in [16].

An unusual feature of this experiment is that all STB infection was natural, with the epidemic caused by a highly diverse *Z. tritici* population that immigrated into the experimental plots via windborne ascospores coming from nearby wheat fields that were treated with fungicides. This local *Z. tritici* population carried sufficient resistance to all fungicides applied in the experimental plots to enable an STB epidemic to develop despite the intensive fungicide treatments. But other wheat diseases common in this region, including leaf rust, stripe rust, stagonospora nodorum blotch, powdery mildew, and tan spot, were practically absent because the fungicides excluded these pathogens [16]. As a result, we were able to obtain a pure-culture read-out of quantitative STB resistance across all 335 wheat cultivars without confounding effects from other diseases. The local weather during the 2015-2016 growing season was cooler and wetter than usual, providing a highly conducive environment for the development of an STB epidemic. At least six asexual reproduction cycles occurred during the most active period of wheat growth between March and July [16]. Other components of STB epidemiology associated with this experiment were already reported [16].

**Figure 1:**
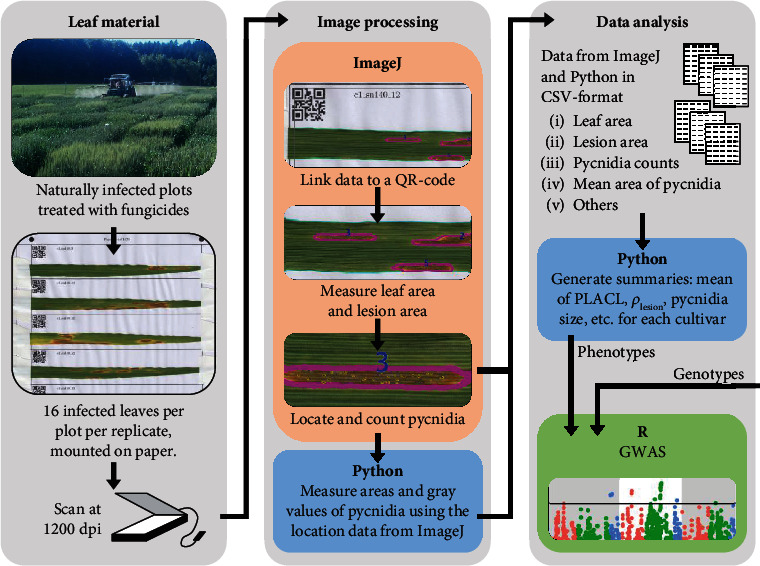
Flow diagram showing steps involved in the acquisition and analysis of phenotype data used in this experiment.

All experimental plots were assessed for STB resistance at two time points, *t*_1_ (20 May 2016, approximately growth stage GS 41 [29]) and *t*_2_ (4 July 2016, approximately GS 75-85) using automated image analysis of 21,420 scanned leaves infected by *Z. tritici* [16, 30]. Nearly sixteen infected leaves collected from the same leaf layer in each plot were mounted on A4 paper and scanned at 1,200 dpi using flatbed scanners as described earlier [16]. The scanned images were analyzed using an ImageJ macro script [16]. Automatically generated outputs of the script included percentage of leaf area covered by lesions (PLACL), average pycnidia density within lesions (*ρ*_lesion_), and average pycnidia darkness (measured using the 256-point gray scale). To measure pycnidia sizes, we developed a Python (version 3.6.7, https://www.python.org/) program based on the determination of contours of constant brightness in the vicinity of each detected pycnidium with the help of the skimage package (version 0.13, https://scikit-image.org/). Each of these STB-associated phenotypes was analyzed separately in the GWAS. The grand means for each phenotype were calculated based on an average of 60 scanned leaves for each wheat cultivar, including both time points and both replicates for each plot (i.e., four measurements of each trait associated with STB resistance). The actual number of leaves analyzed for each cultivar ranged from 31 to 64, with some leaves omitted because of errors made during the collecting, mounting, or scanning processes. Only 35 cultivars had fewer than 55 leaves included in the analysis. The mean values of PLACL and *ρ*_lesion_ were 1/x transformed to better fit a normal distribution, yielding a *P* < 0.01 for the Shapiro-Wilk test after transformation.  Figure 1 illustrates the steps involved in data acquisition and analysis.

The SNP markers used for the GWAS came from the Illumina 90K SNP array (iSelect, San Diego, USA, [31]). The majority of the markers on this array were not useful for our experiment because they were not polymorphic in the GABI panel. The remaining markers were positioned on the IWGSC wheat genome [32] using a BLASTn search with *E* value < 10^−30^. The position with the lowest *E* value was assigned as the marker position. In the case of ties where it was not possible to unequivocally assign a marker to one of the homeologous chromosomes, the markers were omitted. Additional filtering criteria to choose SNPs for the GWAS were a call rate of >95% per marker, >5% minor allele frequency, and identity by state (IBS) < 0.975, using the GenABEL software in the R statistical environment [33]. After filtering, a total of 13,648 high-quality SNP markers were used for the GWAS. Haplotypes were identified using a sliding window of three consecutive SNPs with PLINK [34] and tested using linear regression models. GWAS Manhattan plots were constructed using R (version 3.5.1, [35]) with ggplot2 (version 3.1.0. [36]). Bonferroni thresholds were calculated using *P*/*N* (0.05/13,648) yielding a LOD (-log_10_(*P*)) score of 5.44. The fraction of the phenotypic variance associated with the 26 chromosome intervals at or exceeding the Bonferroni threshold was calculated using linear regression models in R (*lm* function). The adjusted *R*^2^ provided a measure of the proportion of the variance explained.

The coordinates of the 26 intervals exceeding the Bonferroni threshold were plotted onto the Chinese Spring reference genome as described earlier and used to compare the positions of the SNPs associated with the STB resistance traits identified in this analysis with the positions of STB resistance identified in earlier studies [18]. The sequence data of the markers associated with STB resistance in earlier studies were retrieved from GrainGenes (https://wheat.pw.usda.gov/GG3/) and then searched using BLASTn against the IWGSC reference [32] assembly using Unité de Recherche Génomique Info (URGI, https://wheat-urgi.versailles.inra.fr/) for the corresponding chromosome. The position of the best hit was used as the genome position. We then calculated the probability of finding a chance overlap involving at least 16 of the 26 new associations with the previously identified chromosome segments for STB resistance. We treated each new association as an individual point because the new segments were very narrow and calculated the probabilities of overlaps based on a binomial distribution. For each new association, the probability of overlapping with an already known STB resistance interval (which we extended by 5 Mbp at both ends) was 41.2% (i.e., 5.89 Gbp of the 14.30 Gbp genome was included in the previously known intervals, many of which included a large fraction of a chromosome).

Candidate gene identification was based on the gene annotation of the IWGSC v1.0 reference sequence of the wheat landrace Chinese Spring [32]. All high confidence genes in chromosome segments shorter than 5 Mb were identified.

## 3. Results

The three fungicide treatments eliminated all competing fungal pathogens, enabling a mono disease readout of the relative degree of quantitative resistance to STB under the field conditions typically used for intensive wheat production in Europe. All STB infection was natural, with an epidemic resulting from at least six cycles of infection by a diverse *Z. tritici* population that included a high degree of gene and genotype diversity, with infections caused by millions of different *Z. tritici* strains despite the fungicide applications. As an indicator of the pathogen genetic diversity in these plots, genome sequences of 161 *Z. tritici* isolates obtained from 21 of the plots revealed 147 unique genome sequences, with all but two of the identified clones found within the same 1.5 m^2^ plot (Daniel Croll, personal communication). This high level of pathogen diversity was consistent with earlier findings from other naturally infected wheat fields around the world and was expected given that *Z. tritici* populations experience high levels of recombination [11, 35] that enable different fungicide resistance mutations to segregate and reassort into many different genetic backgrounds in natural populations.

The automated analysis pipeline generated phenotypes for 21,420 leaves, with an average of 30 leaves sampled per plot across the two time points. Nearly 37 m^2^ of leaf area was analyzed, with approx. 11 m^2^ scored as damaged by STB. The mean analyzed area for an individual leaf was 17 cm^2^. The percentage of leaf area covered by STB lesions (PLACL) ranged from 0 to 99 with a mean value of 32. The number of pycnidia found on a leaf ranged from 0 to 4,034, with a mean value of 127. The density of pycnidia within STB lesions (*ρ*_lesion_) ranged from 0 to 256 per cm^2^ of lesion, with a mean value of 24. The automatically generated phenotypes included 21,420 measures each of PLACL and *ρ*_lesion_ and 2.7 million measures each of pycnidia size and pycnidia melanization, yielding a total of >5.44 million automatically measured phenotypes that were not prone to human scoring error.

The quantitative measures of STB severity generated by automated image analysis followed the continuous distribution typically associated with quantitative traits [16, 30]. Earlier analyses of relationships among these traits [16] showed that resistance that minimizes host damage (PLACL) was largely independent of resistance that minimizes pathogen reproduction (*ρ*_lesion_). Hence, the GWAS was conducted independently for each trait. In addition to the traits PLACL and *ρ*_lesion_, we measured the average size of pycnidia formed within lesions, which reflects the average size and number of spores contained in each fruiting body [24] (i.e., pycnidia size is an independent indicator of pathogen reproduction), and the average gray value of pycnidia, which reflects the average amount of melanin accumulated in each fruiting body [25, 30]. Our earlier work indicated that pycnidia melanization is on average greater on wheat cultivars with more resistance to STB [24, 30].

**Figure 2:**
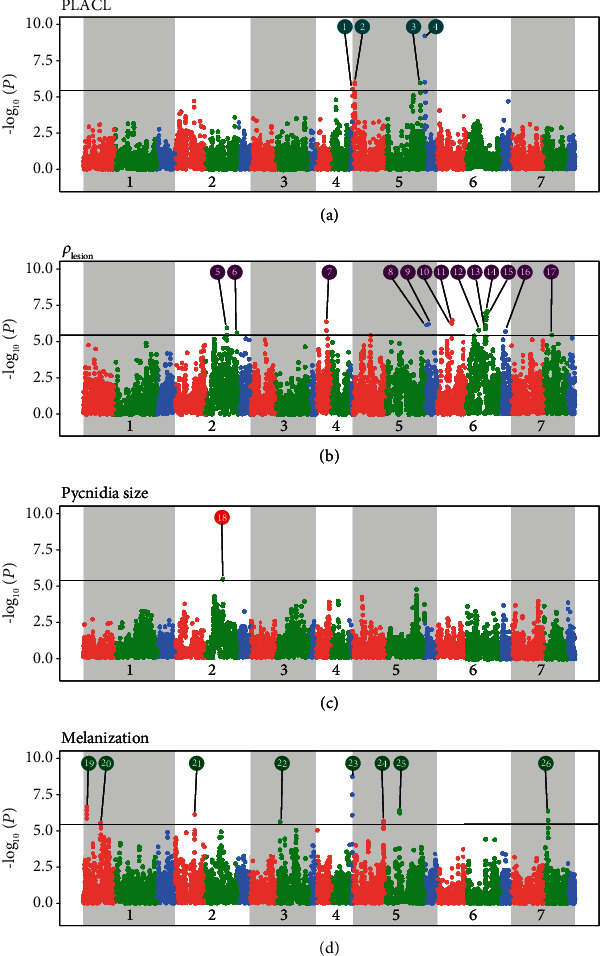
Manhattan plots showing significant SNP markers associated with each trait. The horizontal line indicates the Bonferroni-adjusted significance threshold. The A, B, and D genomes of wheat are shown in red, green, and blue, respectively. SNPs associated with the interval IDs shown in Table 1 are indicated in colored circles. (a) Percentage of leaf area covered by lesions (PLACL) had four significant associations distributed across chromosomes 5A, 5B, and 5D. (b) Density of pycnidia within lesions (*ρ*_lesion_) had 13 significant associations distributed across chromosomes 2B, 4A, 5A, 5D, 6A, 6B, 6D, and 7B. (c) Pycnidia size had a single significant association located on chromosome 2B. (d) Pycnidia melanization had 8 significant associations distributed across chromosomes 1A, 2A, 3B, 4D, 5A, 5B, and 7B.

Manhattan plots for PLACL, *ρ*_lesion_, pycnidia size, and pycnidia gray value revealed the SNPs with the highest associations for each STB resistance trait (Figure 2). A total of 109 SNPs were at or above the Bonferroni threshold across all traits based on the GWAS. Marker-trait associations were calculated using sliding windows including three consecutive SNPs. Among these, 52 haplotypes were at or above the Bonferroni threshold. Further evaluation of the 52 haplotypes revealed overlaps that were combined to produce a nonredundant set of 26 chromosome segments that explained from 1.9% to 10.6% of the overall phenotypic variance associated with each measured trait (Table 1).

For the PLACL trait that reflects the ability of a wheat cultivar to limit the degree of necrosis caused by an STB infection, 14 SNPs identified 4 different genomic positions distributed across chromosomes 5A, 5B, and 5D with LOD scores exceeding 5.5. Interval 4 on 5D had a LOD score of 9.2 and explained 10.3% of the total variance associated with PLACL (Table 1). For the *ρ*_lesion_ trait that reflects the ability of a wheat cultivar to restrict *Z. tritici* reproduction, 51 SNPs identified 13 genomic positions located on chromosomes 2B, 4A, 5D, 6A, 6B, 6D, and 7B, with LOD scores ranging from 5.5 to 7.1. Interval 15 on 6B had the highest LOD score and explained 9.3% of the phenotypic variance associated with *ρ*_lesion_. For the pycnidia size trait, three SNPs located on 2B defined a chromosome interval that surpassed the Bonferroni threshold. Interval 18 explained 5.9% of the total variance associated with pycnidia size. For the pycnidia melanization trait, 36 SNPs defined 8 genomic positions located on chromosomes 1A, 2A, 3B, 4D, 5A, 5B, and 7B. Interval 23 on 4D showed the highest LOD score of 8.7 and explained 10.6% of the total variance associated with pycnidia melanization.

**Figure 3:**
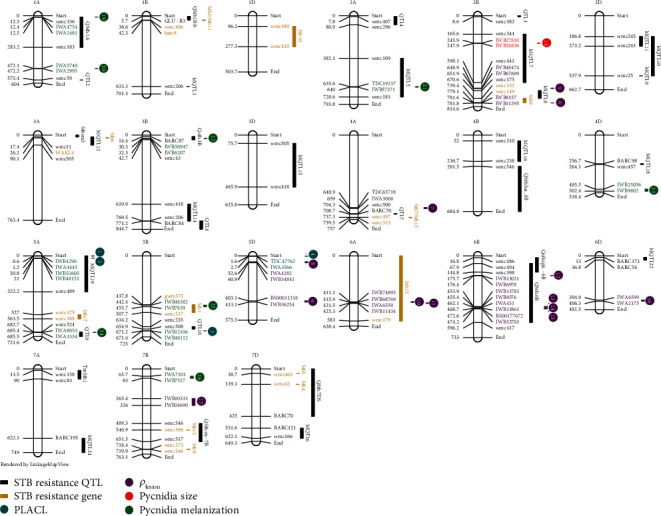
Positions on the Chinese Spring reference genome (IWGSC 2018) of 26 significant GWAS marker-trait associations across four resistance traits compared to positions of previously mapped STB resistance genes [18]. The 26 associations are shown as numbered circles and a bar (95% confidence interval) in cyan for PLACL, purple for *ρ*_lesion_, red for pycnidia size, and green for melanization. Confidence intervals of previously mapped STB resistance loci are shown in yellow bars (STB resistance genes) and black bars (STB resistance QTLs). SNP markers are presented as locus names from GrainGenes (https://wheat.pw.usda.gov/GG3/) for brevity. Markers with the prefix Tdurum_contig were abbreviated to TDC. Only SNP markers with significant associations with STB genes, QTLs, or the four phenotypes included in the GWAS are shown. For each association confidence interval, the first and the last SNP and their positions are shown. Names are colored according to the type of association.

The positions of the 26 chromosome segments identified in this experiment were compared to the positions of mapped STB genes reported in earlier publications (summarized in Figure 1 of [18]).  Figure 3 shows that 16 of the 26 chromosome segments identified in our analyses overlapped with or were very close to genomic regions identified in earlier publications. The probability of finding at least 16 associations within or close to the chromosome intervals previously associated with STB resistance was 2.9%. Ten of the chromosome segments were in chromosomal regions that were not previously associated with STB resistance. Among those, two were associated with PLACL (3, 4), five with *ρ*_lesion_ (7, 8, 9, 16, and 17), and three with pycnidia melanization (20, 23, and 26) (Table 1).

Seventeen of the 26 chromosome segments were smaller than 5 Mb. For these, we identified putative candidate genes responsible for STB resistance based on the wheat reference genome sequence of Chinese Spring [32]. In total, the 17 intervals spanned 26.2 Mb and contained 173 high confidence genes (Supplementary Table 1). There was a significant enrichment (*P* < 0.0001) for genes encoding putative receptor kinases and kinases within these 17 chromosome segments. Receptor kinase genes were recently shown to play major roles in disease resistance in cereals [19, 37, 38], including the *Stb6* gene encoding resistance to STB [19]. Five of the chromosome segments contained four or fewer genes, with three of these segments (19, 20, and 24) associated with pycnidia gray value and two segments (2, 3) associated with PLACL. The smallest chromosome segment (20) encompassed 28 kb on chromosome 1A and contained a single gene in Chinese Spring (TraesCS1A01G277000) encoding a putative solute carrier family 35 member. The 99.7 kb segment 24 on the long arm of chromosome 5A also had a single gene (TraesCS5A01G524800) encoding a putative 4-hydroxy-tetrahydrodipicolinate reductase, a protein involved in lysine biosynthesis. Intervals 2 (chromosome 5A) and 19 (chromosome 1A) had three candidate genes each, of which a putative kinase gene and a putative nucleotide binding site—leucine-rich repeat (NLR)—represent the most obvious candidates as these categories of genes are known to affect disease resistance [39]. Interval 3 contained four candidate genes, all of which were associated with F-box proteins, a class of proteins often associated with plant defense responses [40]. Other candidate genes known to be involved in disease resistance include sugar transporters (associated with PLACL in intervals 1 and 4), superoxide dismutase (associated with pycnidia size in interval 18), an ABC transporter (associated with PLACL in interval 1), and a TCP transcription factor (associated with *ρ*_lesion_ in interval 15).

## 4. Discussion

In a year that was highly conducive for the development of an STB epidemic, we combined a novel automated image analysis tool that could differentiate independent components of STB severity with the high level of fungicide resistance existing in a local Swiss population of *Z. tritici* to make a quantitative comparison of STB resistance across a broad cross section of elite European winter wheat cultivars. GWAS analyses that coupled these quantitative measures of STB resistance with 13,648 SNP markers enabled identification of 109 SNPs on 13 chromosomes that defined 26 chromosome segments highly associated with STB resistance. Because all STB infection in this experiment was natural, including millions of different pathogen genotypes originating from a recombining population, and the growing season was highly conducive to the development of an STB epidemic, we believe that the SNP markers defining the chromosome intervals associated with the highest levels of STB resistance could be especially useful in European breeding programs aimed at increasing overall levels of STB resistance to the *Z. tritici* populations found in Europe.

The 26 chromosome intervals associated with STB resistance ranged from 28 kbp to 60 Mbp in size and were distributed across 13 chromosomes, with individual intervals explaining 1.9% to 10.6% of the phenotypic variance for each trait. Some of the intervals were clustered in the same chromosomal region (e.g., intervals 1 and 2 associated with PLACL on chromosome 5A; intervals 13, 14, and 15 associated with pycnidia gray value on chromosome 6B), but most of the intervals were genetically distant from each other. Sixteen of the intervals were embedded within or located very close to chromosomal regions previously associated with STB resistance, but 10 intervals were in genomic regions that had not been associated with STB resistance. Though the known STB resistance intervals identified in earlier studies were quite large (covering in total ~40% of the entire wheat genome), the probability that at least 16 new intervals would overlap with these known intervals by chance was less than 3%. This finding increases our confidence that our new phenotyping method can identify simultaneously (i.e., in a single experiment) many previously defined STB resistance intervals that had been identified individually in studies involving only one pathogen strain. Particularly notable novel regions were the intervals 4, 8, and 9 located on 5D, a chromosome which had not previously been associated with STB resistance [18], though [27] found some weak associations with STB resistance on this chromosome. There was no overlap between chromosomal segments associated with host damage (PLACL) and pathogen reproduction (pycnidia density or pycnidia size), indicating that these resistance traits were under independent genetic control as hypothesized earlier [16]. Intervals 4, 14, 15, and 23 had LOD scores at or exceeding 7. These are candidate regions for genes encoding broadly based field resistance to STB that may be especially useful against the genetically diverse *Z. tritici* populations in Europe.

The chromosome segments identified in our GWAS are much smaller than the intervals defined in earlier work as shown in Figure 2. For example, *STB15* was previously mapped to a region that includes most of chromosome 6A (~590 Mbp) while we identified two separate chromosome regions (10 and 11) within the *STB15* region that encompass only ~8.5 Mbp. Similarly, *STB1* was mapped to a region that covered ~69.9 Mbp on chromosome 5B while interval 25 covers only ~13 Mbp within this region. The smaller intervals detected in our GWAS reflect the much higher marker density used in our experiment coupled with more accurate knowledge of marker positions coming from the new wheat genome assembly. Other contributors to the small intervals were the more accurate quantitative phenotypes yielding relatively large effect sizes and the haplotype-based GWAS approach that increased the statistical power compared to standard GWAS pipelines.

Seventeen of the 26 chromosome segments identified in the GWAS were less than 5 Mbp in size and contained between 1 and 28 candidate genes annotated in the Chinese Spring reference genome. The 173 genes located in these intervals were significantly enriched for receptor kinases and kinases, including clusters of 6 and 10 kinases found in intervals 18 and 22, respectively. We consider this enrichment to be notable because *Stb6*, the only cloned STB resistance gene, is a receptor kinase [19]. Also notable was our finding that genes encoding receptor kinases are strongly upregulated during infection by all tested strains of *Z. tritici* [38]. Hence, we hypothesize that some of the receptor kinase genes found in these intervals may be responsible for the STB resistance we observed. The interval 4 associated with PLACL explained 10.3% of the overall variance and provided the first report of STB resistance on chromosome 5D. This interval contained 12 genes, including three encoding proteins already shown to affect disease resistance, including an NLR, a S/T protein kinase, and a sugar transporter [39, 41]. We hypothesize that one or more of these genes are responsible for the STB resistance in this chromosome segment. Other interesting candidate genes found in the 17 intervals encode an ABC transporter, a TCP transcription factor, and superoxide dismutase. The *Lr34* gene encoding quantitative resistance to leaf rust and other diseases in wheat was shown to be an ABC transporter [42]. Superoxide dismutases are involved in synthesis of hydrogen peroxide, which was already shown to be involved in wheat's defense response against STB [43]. TCP transcription factors were shown recently to be important components of the signaling pathway involved in systemic acquired resistance [44]. Segment 24, which explained 8% of the variance in pycnidia melanization and lies within the QTL9 region identified in earlier mapping studies, contained a single gene encoding a protein involved in lysine biosynthesis. Recent work on the wheat pathogen *Cochliobolus sativus* showed that lysine was essential for melanin biosynthesis [45] and lysine was recently shown to be essential for virulence in *Z. tritici* [46]. We conclude from this analysis that many of the genes found in the intervals identified in the GWAS are plausible candidates to explain the observed phenotypes associated with STB resistance, but functional validation studies will be needed to confirm whether any of these genes actually play a role in resistance.

Earlier field trials also used association mapping to identify genetic markers associated with STB resistance [27, 47, 48]. In all of these trials, the experimental plots were inoculated with a small number of *Z. tritici* isolates that were sprayed when all wheat genotypes had fully extended flag leaves (i.e., GS > 41) a few weeks before scoring for STB resistance. As a result, the associations identified in those experiments are likely to be strain-specific and represent the outcome of a single cycle of infection based on a high dose of artificially applied blastospore inoculum. Similarly, most experiments that identified STB genes with major effects were based on greenhouse inoculations of seedlings by a single pathogen strain and used disease scores made at a single point in time, leading to identification of genes that encode seedling resistance to the strain used in the experiment. It is now clear that natural field infections of STB are caused by many millions of *Z. tritici* strains, with a different strain occurring on each infected leaf, on average, and with most leaves infected by more than one strain [10, 49]. The significant STB resistance associations identified in our experiment were based on a natural epidemic that included at least six cycles of pycnidiospore infection by a highly diverse population of the pathogen and included two time points during epidemic development. We believe that the STB resistance identified in our experiment is more likely to be broadly applicable under natural field conditions and hence more useful in breeding programs aiming for stable STB resistance.

An important and novel aspect of our experiment was the use of an automated image analysis pipeline for phenotyping that eliminated human scoring bias while generating millions of accurate phenotype datapoints. As is the case for many plant diseases [50], the traditional visual assessment of STB typically generates a single number on a 0-9 scale [51] that tries to integrate the totality of disease in a particular plot, often relative to other plots in the same field or trial. Visual assessments are fast, often requiring less than one minute per plot to produce a measurement, but are prone to variation caused by fatigue, changes in lighting over the course of a day, and differences in opinion among different scorers. The automated image analyses allowed us to simultaneously assess four quantitative phenotypes that could not be accurately measured by the eye. A traditional visual assessment would have generated a total of 4 STB measurements per cultivar to use in the GWAS. Our automated analyses generated an average of over 16,000 STB measurements per cultivar.

The leaf scanning method required significant manual labor (approx. 360 hours in total) to harvest infected leaves, mount them on paper, and scan them on flatbed scanners (Figure 1). A key element of our success was the ability to reproducibly count and measure properties (size and melanization) associated with millions of pycnidia, which measure only 0.5-1.5 mm across. Reliable identification of pycnidia required high-resolution images (1,200 dpi) acquired under uniform, high-intensity lighting, in this case provided by inexpensive flatbed scanners. Further automation of data collection done completely in the field will present new challenges. Flying drones at a height close to the canopy surface may be able to image pycnidia on the top leaf layer, but these drones would need to be coupled with strobe lighting systems and cameras that could produce images with sufficient magnification to reliably identify pycnidia over a significant depth of crop canopy. Ground-based mobile robots would make more sense for data acquisition in row crops such as maize, but wheat fields would be more difficult due to the high plant density. In the near future, we consider it likely that modest amounts of field data (10s-100s of leaves) could be acquired using mobile phone cameras outfitted with clip-on macro lenses or hand-held computers attached to portable single-leaf scanners. We can also envision portable high-throughput conveyor belt leaf scanning systems fit in the bed of a pickup truck that could scan more than 10,000 leaves in a day.

The detailed phenotype data generated by our automated analysis pipeline enabled us to separate different components of STB resistance, in particular allowing us to separate STB resistance that affects host damage (PLACL) from STB resistance that affects pathogen reproduction (*ρ*_lesion_ and pycnidia size). We believe that resistance affecting pathogen reproduction is likely to be more effective in the long run for several reasons, including the following: (1) Our earlier analyses [16] showed that measures of pathogen reproduction (*ρ*_lesion_) early in the growing season were the best predictors of host damage (PLACL) late in the growing season, showing that resistance that reduces pathogen reproduction is likely to minimize yield losses caused by STB. (2) A decrease in pathogen reproduction diminishes the amount of inoculum available to cause new cycles of infection, which will lower the transmission rate (i.e., decrease the basic reproductive number, *R*_0_) during each infection cycle and result in less overall infection by the end of the epidemic. (3) A decrease in pathogen inoculum will lead to a decrease in the pathogen population size, which will decrease the overall genetic diversity and provide fewer opportunities for favorable mutations (e.g., for fungicide resistance or gain of virulence) to emerge. This should lower the overall evolutionary potential of the pathogen population [52].

Recombining the SNP markers associated with the STB resistance intervals identified in this experiment may accelerate breeding efforts aimed at increasing quantitative resistance to STB in European wheat. We showed that resistance affecting leaf damage (PLACL) is genetically distinct from resistance affecting pathogen reproduction (*ρ*_lesion_). We consider it likely that these different resistance phenotypes reflect different underlying mechanisms of STB resistance. We hypothesize that PLACL reflects the additive actions of toxin sensitivity genes that interact with host-specific toxins produced by the pathogen, as shown for *Parastagonospora nodorum* on wheat [53, 54], while pycnidia density reflects the additive actions of quantitative resistance genes that recognize pathogen effectors (e.g., [23]). Under this scenario, breeders should aim to recombine these two forms of resistance into the same genetic background, bringing together different forms of resistance that may be more durable when deployed together than when either mechanism is deployed in isolation. We anticipate that functional analyses of the most compelling candidate genes identified in this experiment will enable us to identify new genes underlying the different STB resistance traits. Our experiment illustrates how high-throughput automated phenotyping can accelerate breeding for quantitative disease resistance.

## Figures and Tables

**Table 1:** Chromosomal intervals defined by 26 significant GWAS associations and the phenotypic variance explained by each association. All chromosome intervals and base pair (BP) positions were defined based on the Chinese Spring reference genome (IWGSC 2018). PLACL = percentage of leaf area covered by lesions; PDL = pycnidia density within lesions (*ρ*_lesion_); MPA = mean pycnidia area (size); PGV = pycnidia gray value; NA = no associations.

Trait	Interval ID	Chromosome	BP start of interval	BP end of interval	Interval size	SNPs in interval	LOD	*R* ^2^	Other known STB R-genes in this region
PLACL	1	5A	644275	1225606	581,331	3	5.51	0.065	MQTL19
PLACL	2	5A	20816445	20998554	182,109	4	5.95	0.068	MQTL19
PLACL	3	5B	671238689	671360289	121,600	3	5.93	0.089	NA
PLACL	4	5D	1612786	2664314	1,051,528	4	9.16	0.103	NA
PDL	5	2B	648912764	651932324	3,019,560	3	5.93	0.078	2 Mbp away from MQTL8
PDL	6	2B	781584349	781830297	245,948	3	5.59	0.085	5 Mbp away from *Stb9*
PDL	7	4A	648893451	658970236	10,076,785	4	6.37	0.021	NA
PDL	8	5D	52615122	60899123	8,284,001	3	6.13	0.044	NA
PDL	9	5D	403075850	413073376	9,997,526	3	6.19	0.066	NA
PDL	10	6A	411095227	415905355	4,810,128	3	6.24	0.019	*Stb15*
PDL	11	6A	421527350	425277806	3,750,456	5	6.47	0.091	*Stb15*
PDL	12	6B	175719208	176403432	684,224	5	5.79	0.073	QStb.6B
PDL	13	6B	453931609	455449000	1,517,391	4	6.03	0.062	QStb.6B
PDL	14	6B	462130236	468736016	6,605,780	8	6.99	0.052	QStb.6B
PDL	15	6B	472609890	474177953	1,568,063	4	7.10	0.093	QStb.6B
PDL	16	6D	394936301	406340008	11,403,707	3	5.70	0.074	NA
PDL	17	7B	265441725	325980844	60,539,119	3	5.46	0.034	NA
MPA	18	2B	245888219	247935045	2,046,826	3	5.50	0.059	MQTL17
PGV	19	1A	12369332	12506454	137,122	6	6.64	0.083	Qstb.1A
PGV	20	1A	472140874	472168930	28,056	4	5.53	0.063	NA
PGV	21	2A	635581134	639988522	4,407,388	3	6.12	0.078	MQTL15
PGV	22	3B	30319666	32286228	1,966,562	3	5.60	0.075	QStb.3B
PGV	23	4D	485524935	502402946	16,878,011	5	8.71	0.106	NA
PGV	24	5A	685438196	685537900	99,704	4	5.67	0.075	QTL9
PGV	25	5B	442374822	455735533	13,360,711	5	6.40	0.096	*Stb1*
PGV	26	7B	65661891	83030127	17,368,236	6	6.34	0.078	NA

## Supplementary Materials

Supplementary Materials Table S1: candidate genes in the chromosome intervals defined by the GWAS.
